# Prospectively Isolated Cancer-Associated CD10^+^ Fibroblasts Have Stronger Interactions with CD133^+^ Colon Cancer Cells than with CD133^−^ Cancer Cells

**DOI:** 10.1371/journal.pone.0012121

**Published:** 2010-08-12

**Authors:** Lin Cui, Kenoki Ohuchida, Kazuhiro Mizumoto, Taiki Moriyama, Manabu Onimaru, Kohei Nakata, Toshinaga Nabae, Takashi Ueki, Norihiro Sato, Yohei Tominaga, Masao Tanaka

**Affiliations:** 1 Department of Surgery and Oncology, Graduate School of Medical Sciences, Kyushu University, Fukuoka, Japan; 2 Department of Advanced Medical Initiatives, Graduate School of Medical Sciences, Kyushu University, Fukuoka, Japan; 3 Department of Cancer Therapy and Research, Graduate School of Medical Sciences, Kyushu University, Fukuoka, Japan; 4 Kyushu University Hospital Cancer Center, Fukuoka, Japan; The University of Hong Kong, Hong Kong

## Abstract

Although CD133 has been reported to be a promising colon cancer stem cell marker, the biological functions of CD133^+^ colon cancer cells remain controversial. In the present study, we investigated the biological differences between CD133^+^ and CD133^−^ colon cancer cells, with a particular focus on their interactions with cancer-associated fibroblasts, especially CD10^+^ fibroblasts. We used 19 primary colon cancer tissues, 30 primary cultures of fibroblasts derived from colon cancer tissues and 6 colon cancer cell lines. We isolated CD133^+^ and CD133^−^ subpopulations from the colon cancer tissues and cultured cells. *In vitro* analyses revealed that the two populations showed similar biological behaviors in their proliferation and chemosensitivity. *In vivo* analyses revealed that CD133^+^ cells showed significantly greater tumor growth than CD133^−^ cells (*P* = 0.007). Moreover, in cocultures with primary fibroblasts derived from colon cancer tissues, CD133^+^ cells exhibited significantly more invasive behaviors than CD133^−^ cells (*P*<0.001), especially in cocultures with CD10^+^ fibroblasts (*P*<0.0001). Further *in vivo* analyses revealed that CD10^+^ fibroblasts enhanced the tumor growth of CD133^+^ cells significantly more than CD10^−^ fibroblasts (*P*<0.05). These data demonstrate that the *in vitro* invasive properties and *in vivo* tumor growth of CD133^+^ colon cancer cells are enhanced in the presence of specific cancer-associated fibroblasts, CD10^+^ fibroblasts, suggesting that the interactions between these specific cell populations have important roles in cancer progression. Therefore, these specific interactions may be promising targets for new colon cancer therapies.

## Introduction

Colorectal cancer is the second leading cause of cancer-related death in the Western world and its incidence is increasing in Asian countries as a result of changes toward a westernized diet [1]. Recently, the concept of cancer stem cells (CSCs) has been focused upon in the biology of colorectal cancer. Colon cancer shows a marked degree of morphological and functional heterogeneity in its cells. Among these cell populations, CSCs in particular have been reported to possess tumorigenic and treatment-resistant activities [2]. Therefore, the isolation and characterization of colon CSCs may help toward the development of novel diagnostic and therapeutic procedures [3].

Human prominin-1 (PROM1, CD133) is a 5-transmembrane glycoprotein of 865 amino acids with a total molecular weight of 120 kDa. It was initially described as a specific surface antigen of human hematopoietic stem cells [4], [5] and a marker for murine neuroepithelial cells and several other embryonic epithelial cells [6]. Although the biological functions of CD133 remain unknown [7], CD133 alone or in combination with other markers is currently used to isolate stem cells from numerous tissues [4], [5], as well as the prostate [8], liver [9] and pancreas [10], [11]. Recently, CD133 was reported to be a CSC marker in colon cancer [3], [12]. Ieta et al. [13] further reported that CD133^+^ cells derived from colon cancer cell lines exhibit higher tumorigenic potential than CD133^−^ cells. However, Shmelkov et al. [14] demonstrated that CD133 is widely expressed by human primary colon cancer epithelial cells, and that both CD133^+^ and CD133^−^ metastatic tumor subpopulations are capable of long-term tumorigenesis *in vivo*. Therefore, the implications of CD133 as a CSC marker remain controversial.

It has been suggested that the interactions between cancer cells and the surrounding stromal fibroblasts have critical roles in tumor invasion and metastasis [15], [16]. Although bulk tumors are known to consist of heterogeneous cancer cells with different proliferation, invasion and metastasis activities, there are no reports focusing on the heterogeneity of cancer-associated fibroblasts. In most previous studies [17], [18], [19], [20], only two or three primary cultures of cancer-associated fibroblasts have been used for investigations. Therefore, for a better understanding of cancer cell-stromal cell interactions, it is important to focus on the heterogeneity of cancer-associated fibroblasts, similar to the heterogeneity of cancer cells, such as CSCs.

In the present study, we investigated the biological implications of CD133^+^ colon cancer cells by analyzing CD133 expression in human primary colon cancer tissues and evaluating the biological behaviors of CD133^+^ and CD133^−^ cells derived from colon cancer cell lines *in vitro* and *in vivo*. We found significant differences in invasiveness between these two populations in cocultures with primary fibroblasts derived from colon cancer tissues, especially in cocultures with fibroblasts positive for CD10, a membrane metalloendopeptidase. Taken together, these data suggest that CD133^+^ colon cancer cells are more invasive in the presence of specific cancer-associated fibroblasts, especially CD10^+^ fibroblasts, and that these cell populations may be promising targets for inhibiting metastasis and tumor recurrence.

## Results

### CD133 expression in surgically resected primary colon cancer tissues

To date, inconsistent data have been reported regarding the CD133^+^ and CD133^−^ cell populations in primary colon cancer tissues [3], [14]. We investigated the percentages of the CD133^+^ and CD133^−^ cell populations in primary colon cancer tissues by flow cytometry. We used 26 primary colon cancer tissues and 24 normal colon epithelial tissues from 26 patients. We analyzed the CD133^+^ populations in these colon tissues after excluding CD45^+^ and CD31^+^ cells. All the colon cancer samples contained both CD133^+^ and CD133^−^ cells (Table 1; CD133^+^ cells: 2–54%). We also detected CD133^+^ cells at 0–5% in normal colon tissues. To evaluate the clinical implications of CD133 expression, we investigated the correlations between CD133 expression and the clinicopathological findings, such as tumor grade, Dukes classification, lymph node metastasis, lymphatic invasion and venous invasion (Table 2). We found significant correlations between CD133 expression and Dukes classification (*P* = 0.010) and between CD133 expression and lymph node metastasis (*P* = 0.023).

**Table 1:** CD133, CD10 and other cell surface marker in colon cancer and normal tissues.

Case	Age/Sex	Site	Grade	Dukes	CD133	CD24	CXCR4	C-Kit	CD44	CD10	CD105	CD54
					T/N	T/N	T/N	T/N	T/N	T/N	T/N	T/N
**1**	58/M	Sigmoid	G2	C	2/-	-/-	-/-	-/-	-/-	-/-	-/-	-/-
**2**	72/M	Sigmoid	G1	B	10/-	-/-	-/-	-/-	14/-	-/-	-/-	-/-
**3**	64/F	Right	G1	B	14/-	-/-	-/-	-/-	91/34	-/-	-/-	-/-
**4**	63/F	Right	G2	C	25/2	-/-	-/-	-/-	22/14	2/3	-/-	-/-
**5**	59/F	Right	G2	C	54/5	-/-	-/-	-/-	10/14	10/8	27/7	-/-
**6**	56/M	Right	G2	B	6/5	-/-	-/-	-/-	16/36	-/-	-/-	-/-
**7**	58/F	Right	G1	C	4/0	-/-	-/-	-/-	12/8	-/-	-/-	-/-
**8**	72/M	Sigmoid	G1	B	20/1	-/-	4/15	-/-	7/21	-/-	-/-	-/-
**9**	64/M	Left	G1	B	11/0	-/-	7/9	-/-	7/2	-/-	-/-	-/-
**10**	60/F	Sigmoid	G2	C	2/0	-/-	-/-	-/-	14/4	3/-	2/-	-/-
**11**	53/M	Right	G1	C	21/0	-/-	-/-	6/2	4/3	6/2	4/3	-/-
**12**	88/F	Sigmoid	G2	B	5/1	-/-	-/-	-/2	7/2	10/4	4/3	-/-
**13**	59/M	Left	G1	C	27/3	-/-	-/-	-/-	6/7	7/4	9/7	-/-
**14**	61/M	Left	G1	B	2/0	4/2	2/2	0.4/0.5	5/3	0.4/0.2	1/0.5	1.5/1
**15**	63/F	Right	G2	C	24/1	78/6	72/5	8/1	23/3	25/1	7/1	22/3
**16**	66/M	Sigmoid	G1	B	13/1	39/8	16/9	2/2	4/1	7/1	3/1	9/1
**17**	83/M	Right	G1	B	27/0	41/4	27/6	7/1	6/3	3/1	4/0.4	7/4
**18**	59/M	Right	G2	B	17/3	13/26	15/35	6/19	60/37	17/6	19/10	26/8
**19**	68/M	Left	G1	B	17/2	22/27	18/25	5/11	69/47	1/5	18/7	4/5
**20**	80/F	Right	G1	B	10/1	13/5	16/14	1/3	57/12	3/1	1.9/2	64/1
**21**	78/F	Right	G1	B	5/1	16/8	12/13	2/1.8	15/7	4/3	1.3/1	10/8
**22**	39/F	Sigmoid	G1	C	21/3	30/14	23/21	2/3	17/12	16/1	4/2	24/4
**23**	75/M	Left	G2	B	29/0	43/3	17/4	8/2	27/5	12/1	8/2	24/3
**24**	65/F	Right	G2	C	5/0	20/4	13/4	2/2	12/5	6/2	1/1	12/3
**25**	72/F	Sigmoid	G2	B	12/5	47/3	12/5	2/1	26/18	2/2	3/1	12/4
**26**	75/F	Sigmoid	G2	B	5/2	6/4	5/3	3/3	13/7	3/5	19/10	9/3

*Flow cytometry analysis for 26 patients. In table numeral is percentage.

**Table 2:** Relation between CD133 or CD10 expression and clinicopathological characteristics in colon adenocarcinoma.

		CD133 expression				CD10 expression			
	Variable	High (≧21%, n = 8)	Low (<21%, n = 18)	High rate	*P* value	High (≧3.4%, n = 12)	Low (<3.4%, n = 7)	High rate	*P* value
Age (yr)									
	<66	6	9	0.400	0.225	8	2	0.800	0.105
	≧66	2	9	0.182		4	5	0.444	
Gender									
	Male	4	9	0.308	1	5	3	0.625	0.926
	Female	4	9	0.308		7	4	0.636	
Grade									
	G1	4	10	0.286	0.793	5	4	0.556	0.514
	G2	4	8	0.333		7	3	0.700	
Dukes									
	B	2	14	0.125	0.010*	5	6	0.455	0.051
	C	6	4	0.600		7	1	0.875	
Lymph									
node	Negative	2	13	0.133	0.023*	5	6	0.455	0.051
metastasis	Positive	6	5	0.545		7	1	0.875	
Lymphatic									
invasion	0	4	13	0.235	0.277	7	5	0.636	0.565
	1, 2	4	5	0.444		5	2	0.714	
Venous									
invasion	0	4	12	0.267	0.423	10	3	0.833	0.068
	1, 2, 3	4	6	0.400		2	4	0.333	

### Comparisons of malignant behaviors between sorted CD133^+^ and CD133^−^ colon cancer cells

**Figure 1:**
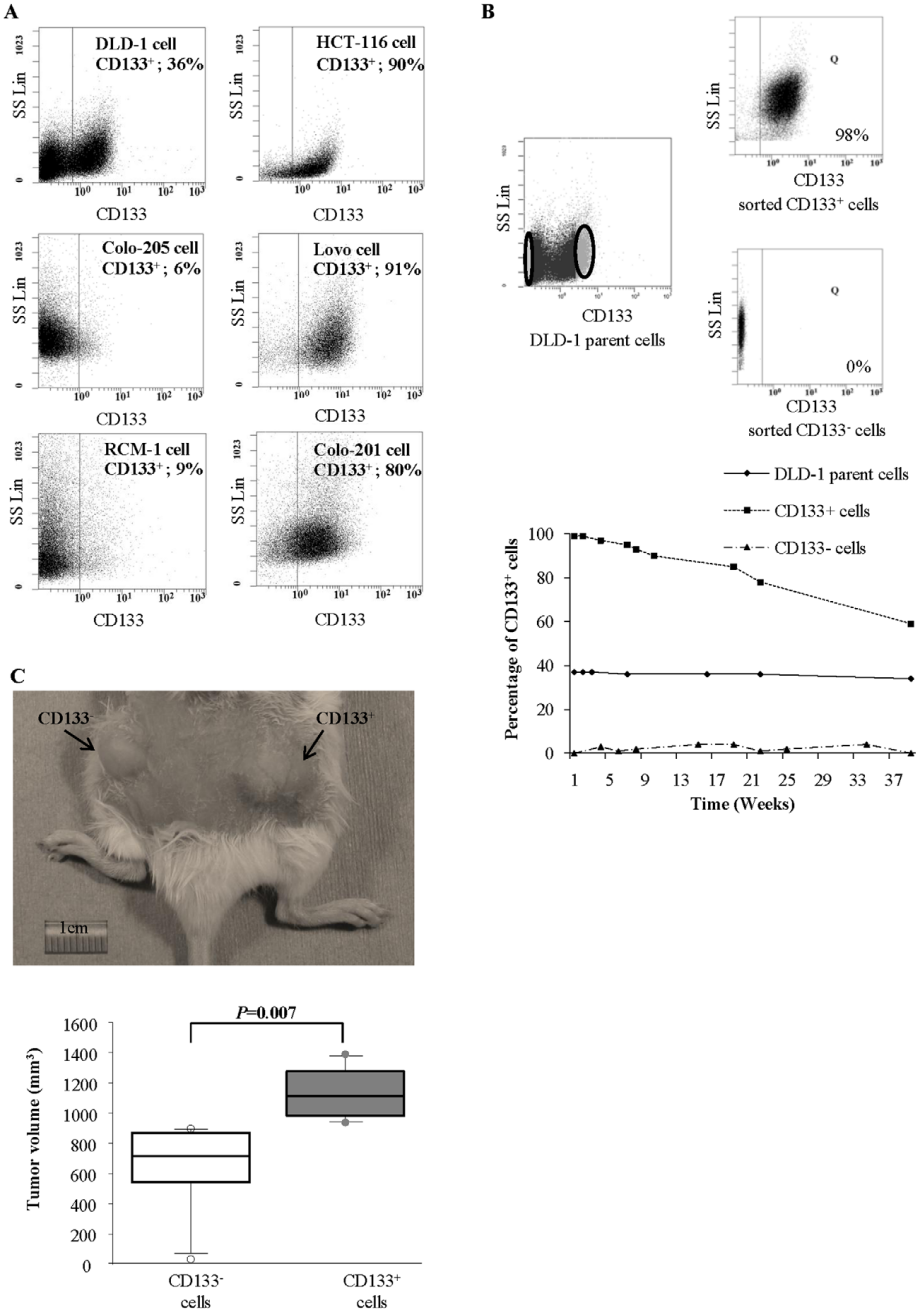
Biological behaviors of CD133^+^ colon cancer cells *in vitro* and *in vivo*. (**A**) CD133^+^ populations in a panel of human colon cancer cell lines. Flow cytometry analyses were performed to investigate the CD133^+^ populations in 6 colon cancer cell lines. (**B**) Analysis of the CD133^+^ population in parental DLD-1 cells and reanalyses of sorted CD133^+^ cells (98%) and CD133^−^ cells (100%) (upper panels). The time-dependent changes in the CD133^+^ populations in unsorted parental and sorted CD133^+^ and CD133^−^ DLD-1 cells are also shown (lower panel). The sorted CD133^+^ cells show significant time-dependent changes in the CD133^+^ and CD133^−^ populations during long-time culture, while the percentages of the CD133^+^ and CD133^−^ populations do not change in the parental cells and sorted CD133^−^ cells. (**C**) Tumor growth was evaluated at 1 month after injection of 5×10^6^ CD133^+^ or CD133^−^ DLD-1 cells into 6 SCID mice, respectively. Representative photographs are shown in the upper panel (left: CD133^−^ cell-derived tumor; right: CD133^+^ cell-derived tumor). Scale bar = 1 cm. The CD133^+^ cell-derived tumors are significantly larger than the CD133^−^ cell-derived tumors (*P* = 0.007; lower panel). Data represent means ± SD.

Using flow cytometry, we examined the presence of CD133^+^ populations in 6 colon cancer cell lines. We found that all the cell lines possessed CD133^+^ cells (6–91%; Figure 1A). In particular, we detected two distinct populations, one consisting of only CD133^+^ cells and the other consisting of only CD133^−^ cells, in DLD-1 cells, while the other cell lines appeared to possess one population consisting of both CD133^+^ and CD133^−^ cells. We sorted DLD-1 cells on the basis of CD133 expression and reanalyses showed effective selection (CD133^+^: 98%; CD133^−^: 100%; Figure 1B). Subsequently, we investigated the time-dependent changes in CD133 expression on unsorted DLD-1 parental cells and sorted CD133^+^ and CD133^−^ cells. As shown in Figure 1B, sorted CD133^+^ cells showed significant time-dependent changes in the CD133^+^ and CD133^−^ populations during long-time culture, while the percentages of the CD133^+^ and CD133^−^ populations did not change in the parental cells and sorted CD133^−^ cells. Next, to compare the biological behaviors of sorted CD133^+^ and CD133^−^ cells, we used DLD-1 and HCT116 cells and investigated their cell proliferation, colony formation ability and chemoresistance to 5-fluorouracil (5-Fu) and doxorubicin (DOX). We found no significant differences in these cell behaviors between CD133^+^ and CD133^−^ cells (Figures S1, S2, S3, S4).

To evaluate the tumorigenic potential and *in vivo* tumor growth of CD133^+^ and CD133^−^ DLD-1 cells, we transplanted serial numbers of CD133^+^ and CD133^−^ cells into severe combined immunodeficiency (SCID) mice. Although there was a trend toward earlier detection of tumor formation in mice injected with CD133^+^ cells compared with mice injected with CD133^−^ cells, the differences were not significant (Table 3). At 10 weeks after transplantation, injection of more than 5×10^6^ CD133^+^ or CD133^−^ cells generated visible tumors in all mice. However, when the tumor sizes were compared, the CD133^+^ cell-derived tumors were significantly larger than the CD133^−^ cell-derived tumors (*P* = 0.007; Figure 1C).

**Table 3:** Tumorigenic potential of CD133^+^/CD133^−^ cells derived from DLD-1 cells.

		No. of mice with tumor formation		
		(Tumor volume >3cm^3^)		
	No. of injected cells	6 Weeks	8 Weeks	10 Weeks
CD133^+^	4×10^4^	1/4	1/4	1/4
	2×10^5^	1/5	1/5	1/5
	1×10^6^	3/5	5/5	
	5×10^6^	4/4		
CD133^−^	4×10^4^	0/4	0/4	1/4
	2×10^5^	0/5	0/5	1/5
	1×10^6^	3/5	4/5	5/5
	5×10^6^	4/4		

### Cancer-associated fibroblasts enhance the invasiveness of CD133^+^ cells more effectively than that of CD133^−^ cells

**Figure 2:**
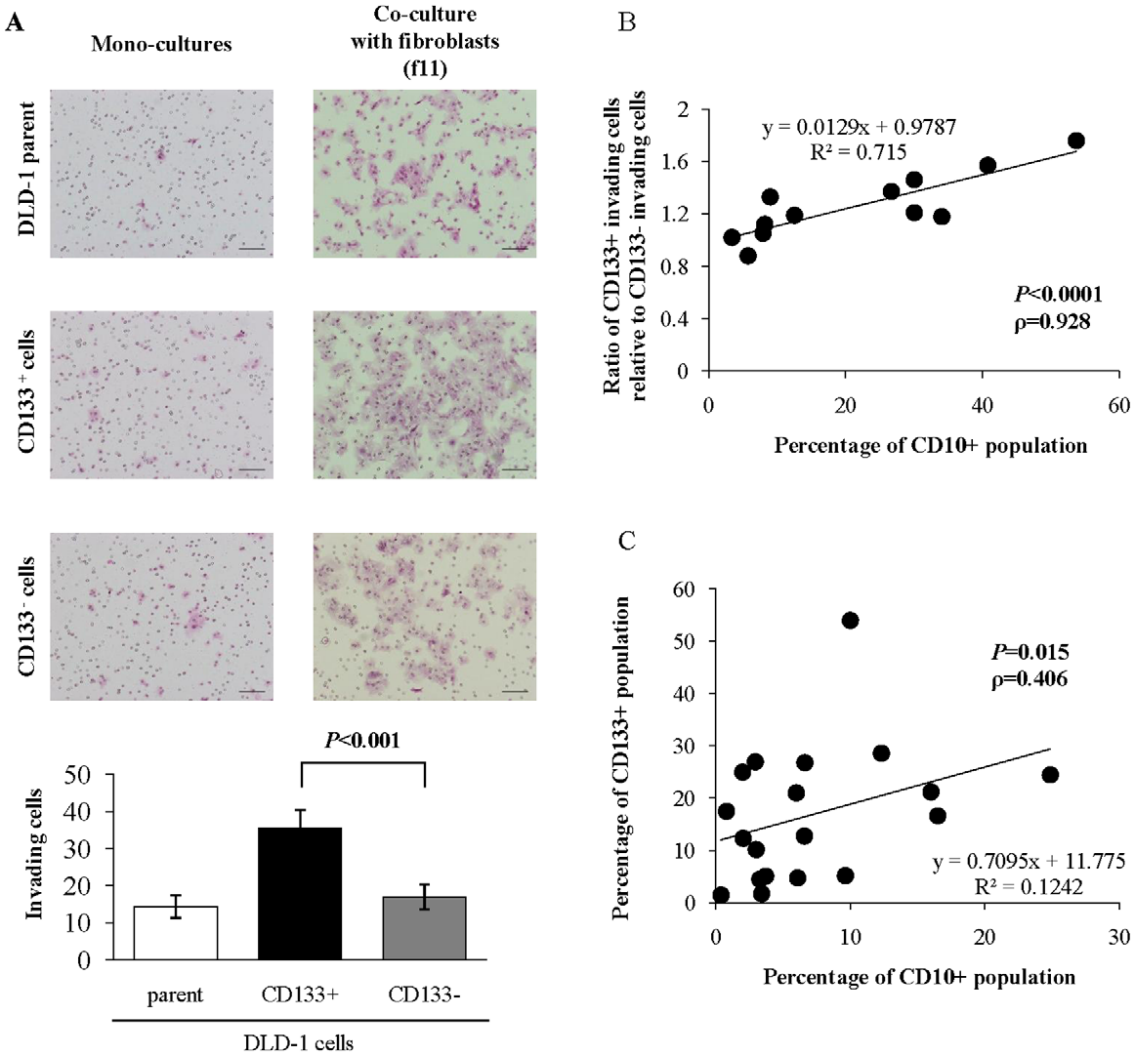
Cancer-associated fibroblasts enhance the malignant behaviors of CD133^+^ colon cancer cells and the percentage of the CD10^+^ population in fibroblasts is correlated with colon cancer invasion. (**A**) Invading cells were measured after 48 h of monoculture or coculture with cancer-associated fibroblasts (f11). CD133^+^ cells cocultured with cancer-associated fibroblasts show significantly greater invasiveness than CD133^−^ cells and parental cells (*P*<0.001). Scale bars = 100 µm. (**B**) The percentages of the CD10^+^ cell population and the activities of these cells to induce the invasion of cocultured colon cancer cells were investigated in 12 primary cultures of fibroblasts. There is a significant positive correlation between the enhancement of CD133^+^ cell invasiveness and the percentage of the CD10^+^ cell population (*P*<0.0001). (**C**) Flow cytometry analyses were performed to investigate the CD10^+^ populations in primary colon cancer tissues. There is a significant correlation between the percentages of the CD133^+^ and CD10^+^ populations in colon cancer tissues (*P* = 0.015).

To elucidate the factors causing the differences in the *in vivo* tumor growth between CD133^+^ and CD133^−^ cells, we investigated the effects of cocultures with cancer-associated fibroblasts on the invasiveness of these cells. We found that there was no difference in invasiveness between CD133^+^ and CD133^−^ cells when the cells were cultured alone (Figure 2A). In contrast, when the cells were cocultured with cancer-associated fibroblasts, CD133^+^ cells showed significantly greater invasiveness than CD133^−^ cells and the parental cells (*P*<0.001 for both; Figure 2A). We examined the effects of 12 primary cultures of fibroblasts on the invasiveness of CD133^+^ cells, and found that the effects of the cocultured cells on CD133^+^ cell invasion varied from 0.88-fold to 1.76-fold (Table 4).

**Table 4:** Expression profiling of CD10, CD105, CD44 and CD54 in primary cultures of fibroblasts.

	CD10	CD105	CD44	CD54	Invasion
					(CD133^+^/CD133^−^)
f1	26.67	59.55	94.22		1.37
f2	12.54	41.25	96.90	18.83	1.19
f3	5.76	85.17	96.65		0.88
f4-1	3.40	96.08	99.08		1.02
f4-2	7.98	75.42	97.86		1.05
f4-3	3.00	86.77	99.15		
f5	30.06	70.18	99.66		1.21
f6	30.06	70.18	99.22	5.11	1.46
f7	0.87	96.14	98.62		
f9	8.23	96.13	99.47	6.97	1.12
f10	9.47	84.52	96.50		
f11	53.71	59.04	98.78	26.50	1.76
f12	8.12	88.45	99.94	8.65	
f13	8.98	87.03	93.81	7.17	1.33
f14	6.86	82.49	97.69		
f15	73.33	1.32	96.64	7.15	
f16	40.78	59.07	96.24	24.42	1.57
f17	5.89	86.41	99.96	82.34	1.18
f18	56.24	42.40	98.55		
f19	5.37	88.78	99.91	86.23	
f20	2.34	78.17	99.56	79.99	
f21	0.44	95.76	99.70	85.54	
f22	0.39	85.00	97.73	20.86	
f23	0.53	66.90	98.39	13.33	
f24	18.02	64.62	99.75	71.46	
f25	2.50	82.32	99.77	82.03	
f26	3.86	83.93	99.78	94.02	
f27	37.35	49.40	99.89	71.31	
f28	36.52	60.44	99.75	72.78	
f29	13.88	63.39	98.21	63.15	
f31	25.16	52.65	89.74	13.44	
f32	6.34	90.40	99.28	72.77	

### Correlations between the percentages of cell surface marker-positive populations in cancer-associated fibroblasts and their enhancement of cancer invasion

To characterize the primary cultures of cancer-associated fibroblasts, we examined the expressions of the stromal cell-associated surface markers CD10, CD105, CD44 and CD54 on 32 primary cultures of fibroblasts by flow cytometry (Table 4). We then investigated the correlations between the enhancement of CD133^+^ cell invasiveness and the percentages of positive populations for these surface markers. We found a significant correlation between the enhancement of CD133^+^ cell invasiveness and the percentage of the CD10^+^ population (*P*<0.0001; ρ = 0.928; Figure 2B; Table 5). As shown in Table 4, the percentage of the CD10^+^ population in primary cultures of cancer-associated fibroblasts established from colon tumors ranged from 0.39% to 73.33%. We also found a significant inverse correlation between the percentages of the CD10^+^ and CD105^+^ populations (*P*<0.0001; Table 5), as well as a significant inverse correlation between the enhancement of CD133^+^ cell invasiveness and the percentage of the CD105^+^ population (*P* = 0.0268; Table 5).

**Table 5:** Correlation by CD10, CD105, CD44 and invasion.

			Correlation Coefficient (ρ)	*P* value (Prob>|ρ|)
CD10	&	CD105	−0.7191	<.0001*
CD44	&	CD10	−0.3150	0.0844
CD44	&	CD105	0.5000	0.0453*
Invasion	&	CD10	0.9282	<.0001*
Invasion	&	CD105	−0.6340	0.0268*
Invasion	&	CD44	−0.2028	0.5273

Next, we investigated CD10 expression in surgically resected primary tissues and found that 0.4–25% of the cells derived from colon cancer tissues were CD10^+^ (Table 1). We then examined the correlations between the percentage of the CD10^+^ population and the clinicopathological findings. We found trends toward positive correlations between the percentage of the CD10^+^ population and Dukes classification and between the percentage of the CD10^+^ population and lymph node metastasis, although they were not statistically significant (Table 2). We also investigated the correlations among the cell surface markers in the same patients and found a significant correlation between the percentages of the CD133^+^ and CD10^+^ populations (*P* = 0.015; ρ = 0.4063; Figure 2C).

### CD10^+^ fibroblasts enhance the *in vitro* invasion and *in vivo* tumor growth of CD133^+^ cancer cells

**Figure 3:**
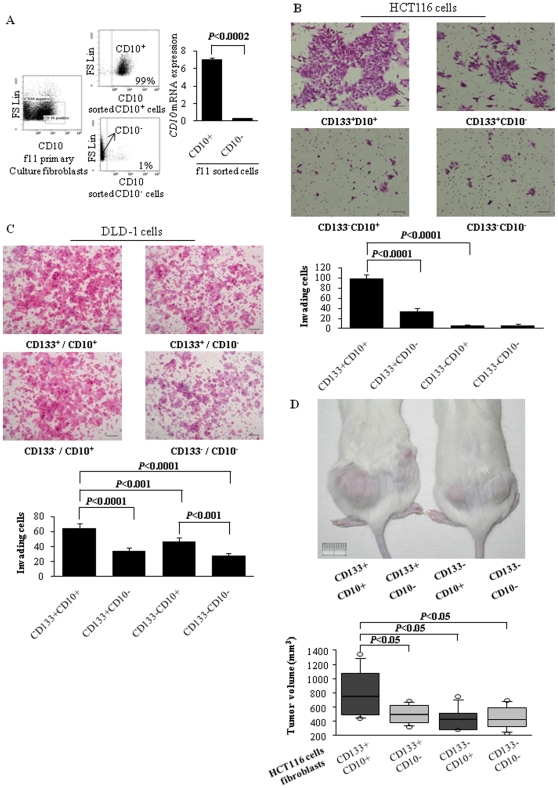
CD10^+^ fibroblasts enhance the *in vitro* invasion and *in vivo* tumor growth of CD133^+^ cancer cells. (**A**) Analysis of primary cultures of colon cancer-associated fibroblasts (left panel) and reanalyses of sorted CD10^+^ cells (middle upper panel) and CD10^−^ cells (middle lower panel). The *CD10* mRNA levels in the sorted CD10^+^ and CD10^−^ fibroblasts were measured by qRT-PCR (right panel). (**B, C**) The invasiveness of CD133^+^ and CD133^−^ cancer cells cocultured with CD10^+^ or CD10^−^ fibroblasts was evaluated. Representative photographs are shown (B, HCT116 cells; C, DLD-1 cells). Scale bars = 100 µm. CD10^+^ fibroblasts enhance the invasiveness of CD133^+^ cancer cells significantly more than CD10^−^ fibroblasts (*P*<0.0001). (**D**) The effects of CD10^+^ and CD10^−^ fibroblasts on *in vivo* tumor growth were evaluated at 4 weeks after cotransplantation of CD133^+^ or CD133^−^ HCT116 cancer cells and CD10^+^ or CD10^−^ fibroblasts into SCID mice (*n* = 6 for each group). Representative photographs are shown (upper panel). Scale bar = 1 cm. CD10^+^ fibroblasts enhance the tumor growth of CD133^+^ HCT116 cancer cells significantly more than CD10^−^ fibroblasts (*P*<0.05; lower panel). Data represent means ± SD.

To investigate the functional differences between CD10^+^ and CD10^−^ fibroblasts, we sorted CD10^+^ and CD10^−^ fibroblasts (Figure 3A) from primary cultures of cancer-associated fibroblasts. The levels of *CD10* mRNA in these two populations were consistent with the levels of CD10 cell surface protein expression (Figure 3A). Using these two populations of fibroblasts, we investigated their effects on the invasiveness of CD133^+^ and CD133^−^ cancer cells. CD10^+^ fibroblasts enhanced the invasiveness of CD133^+^ cancer cells significantly more than CD10^−^ fibroblasts (*P*<0.0001; HCT116 cells, Figure 3B; DLD-1 cells, Figure 3C). CD10^+^ fibroblasts also slightly enhanced the invasiveness of CD133^−^ cancer cells more than CD10^−^ fibroblasts, but significantly less than their effect on the invasiveness of CD133^+^ cancer cells (*P*<0.001; Figure 3C). To evaluate the effects of CD10^+^ and CD10^−^ fibroblasts on *in vivo* tumor growth, we cotransplanted CD133^+^ or CD133^−^ HCT116 cancer cells and CD10^+^ or CD10^−^ fibroblasts into SCID mice. CD10^+^ fibroblasts enhanced the tumor growth of CD133^+^ cancer cells significantly more than CD10^−^ fibroblasts (*P*<0.05; Figure 3D).

### Effect of CD10 knockdown in CD10^+^ fibroblasts on the invasion of cocultured colon cancer cells

**Figure 4:**
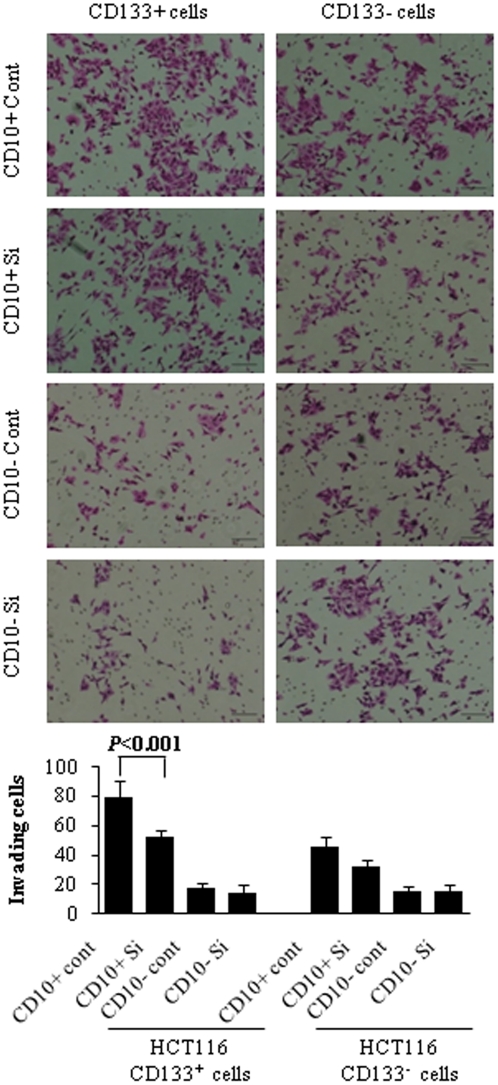
Invasiveness of CD133^+^ and CD133^−^ HCT116 cells cocultured with fibroblasts transfected with control or *CD10*-targeting siRNAs. Knockdown of CD10 expression in CD10^+^ fibroblasts remarkably reduces the invasiveness of cocultured CD133^+^ HCT116 cancer cells (*P*<0.001), but has a limited effect on the invasiveness of CD133^−^ cancer cells. Representative photographs (upper panels) and graphs (lower panel) are shown. Scale bars = 100 µm.

To examine whether CD10 protein in fibroblasts functionally contributes to the enhancement of invasion of cocultured cancer cells, fibroblasts transfected with *CD10*-targeting small interfering RNAs (siRNAs) (siRNA-1 and siRNA-2) or a control siRNA were used for invasion assays. Both of the *CD10*-targeting siRNAs inhibited 90% of *CD10* mRNA expression. Knockdown of CD10 expression in CD10^+^ fibroblasts remarkably reduced the invasiveness of cocultured CD133^+^ HCT116 cancer cells (*P*<0.001; Figure 4) but had a limited effect on the invasiveness of CD133^−^ cancer cells.

### Differential expression profiling of CD10^+^ and CD10^−^ fibroblasts

To elucidate the key molecules involved in the CD10^+^ fibroblast-mediated enhancement of the *in vitro* invasion and *in vivo* tumor growth of CD133^+^ colon cancer cells, we performed microarray analyses using CD10^+^ and CD10^−^ primary-cultured fibroblasts derived from colon tumors. Comparisons of the microarray data between CD10^+^ and CD10^−^ primary-cultured fibroblasts identified 20 genes that were upregulated by >2-fold in CD10^+^ fibroblasts (Table S1). To validate these microarray data, qRT-PCR was performed. We selected *ALDH1A1*, *GPNMB*, *MMP3* and *IGFBP2* from the genes listed in Table S1 because these genes have been reported to be involved in cancer cell invasion and colonic stem cells was also reported to express ALDH1 [21], [22], [23], [24], [25]. The validated data were consistent with the microarray data (Figure S5).

To evaluate the effects of *CD10* knockdown on the expressions of the four invasion-associated genes (*MMP3*, *ALDH1A1*, *GPNMB* and *IGFBP2*) in CD10^+^ fibroblasts, CD10^+^ fibroblasts were transfected with *CD10*-targeting or control siRNAs, and the expressions of the four invasion-associated genes were assessed. There were no significant changes in the expressions of these four genes (Figure S6).

## Discussion

In the present study, our *in vitro* experiments revealed that CD10^+^ fibroblasts derived from colon cancer tissues significantly enhanced the invasion of CD133^+^ colon cancer cells compared with CD10^−^ fibroblasts. We also found that knockdown of CD10 in CD10^+^ fibroblasts partially reduced the invasiveness of cocultured CD133^+^ colon cancer cells. Furthermore, our *in vivo* analyses demonstrated that cotransplantation of CD10^+^ fibroblasts significantly increased the tumor growth of CD133^+^ colon cancer cells compared with cotransplantation of CD10^−^ fibroblasts. Taken together, these data suggest that a specific subset of colon cancer cells, CD133^+^ cells, has important interactions with a specific subset of cancer-associated fibroblasts, CD10^+^ fibroblasts. This is the first report to describe cancer cell-stromal cell interactions between prospectively sorted specific subsets of colon cancer cells and cancer-associated fibroblasts.

CSCs have been isolated from colon cancer on the basis of their expression of the cell surface marker CD133 [3], [12], [26]. Although several investigators have recently published reports about CD133^+^ colon cancer cells [3], [12], [26], there were inconsistent data regarding the tumor-initiating ability of CD133^+^ colon cancer cells. In the present study, we found tumorigenic potential in both CD133^+^ and CD133^−^ colon cancer cells, consistent with the results of Shmelkow et al. [14]. Furthermore, we found no significant differences between these two cell populations in their *in vitro* cell proliferation, colony formation and chemoresistance. However, the present *in vivo* analyses revealed that CD133^+^ cells generated significantly larger tumors than CD133^−^ cells. To understand the implications of the inconsistent results between the *in vitro* and *in vivo* experiments, we focused on cancer cell-stromal cell interactions, which are known to have crucial roles in cancer progression [27]. In cocultures with several primary cultures of cancer-associated fibroblasts, the invasiveness of CD133^+^ colon cancer cells was significantly increased compared with that of CD133^−^ cells, while primary cultures of other cancer-associated fibroblasts did not seem to have such potential to enhance the invasiveness of cancer cells. Therefore, we characterized these primary cultures of cancer-associated fibroblasts, and found that CD10^+^ fibroblasts played a significant role in the enhancement of CD133^+^ cancer cell invasiveness in *in vitro* and *in vivo* experiments.

In the present study, we used CD133^+^ and CD133^−^ cells isolated from cultured cell lines because we were unable to obtain reproducible results for tumorigenicity assays and coculture experiments when we used CD133^+^ and CD133^−^ cells isolated from patients' tumors in a preliminary study. However, it is important to investigate the biological behaviors of CD133^+^ and CD133^−^ subpopulations isolated from patients' tumors. Therefore, further examinations are required to clarify the relationship between primary tumor-derived CD133^+^ cancer cells and CD10^+^ fibroblasts. However, we need to improve our methods for primary cultures of cancer cells before such examinations can be performed. In addition, we used subcutaneous models in the present study because of difficulties encountered in the assessment of tumor size in orthotopic models. However, considering the importance of the tissue microenvironment, studies using orthotopic models should be performed as the next step.

CD10 is a 90–100-kDa cell surface zinc-dependent metalloprotease that is widely expressed by the cells in various tissues, such as lymphoid progenitor cells, myoepithelial cells and stromal cells of the normal bone marrow and endometrium [28], [29], [30], [31]. In addition, CD10 was reported to be a marker for categorizing acute leukemias and subclassifying malignant lymphomas [32]. Recent immunohistochemical studies revealed that the frequency of positive CD10 stromal expression in tumors was significantly correlated with the prognosis of patients with breast cancers [18] as well as the invasion and metastasis of gastric cancers [19]. Ogawa et al. [31] performed an immunohistochemical study of colorectal cancer and reported that expression of CD10 in stromal cells was more frequently detected in invasive tumors than in non-invasive tumors. The findings of these previous immunohistochemical studies are consistent with those of our present functional study.

The present results demonstrated that knockdown of CD10 expression in CD10^+^ fibroblasts partially, but significantly, decreased the invasive ability of cocultured CD133^+^ colon cancer cells. Our microarray data identified several genes that are involved in the invasion of cancer cells, but knockdown of CD10 expression did not affect the expressions of these genes. The data may suggest that CD10^+^ fibroblasts have both CD10-dependent and CD10-independent mechanisms to promote the invasion of cocultured CD133^+^ colon cancer cells, although further examinations are required to elucidate the detailed mechanisms of these interactions.

In conclusion, the present functional analyses suggest that interactions between CD133^+^ colon cancer cells and cancer-associated CD10^+^ fibroblasts have important roles in colon cancer progression. These interactions may be promising targets for colon cancer therapies.

## Materials and Methods

### Ethics statement

The study was approved by the Ethics Committee of Kyushu University (approval number, 19-13) and conducted according to the Ethical Guidelines for Human Genome/Gene Research enacted by the Japanese Government. All the patients provided signed informed consent approving the use of their tissues for unspecified research purposes. For all experiments involving mice, the animals were housed in laminar-flow cabinets under specific pathogen-free conditions in facilities approved by Kyushu University (approval number, A020-018-0).

### Cells and reagents

Human colon cancer cell lines (DLD-1, RCM1 and Colo201) were purchased from the Japanese Collection of Research Bioresources (Osaka, Japan). The LOVO and Colo205 cell lines were obtained from RIKEN (Tsukuba, Japan) and the HCT116 cell line was obtained from the American Type Culture Collection (Manassas, VA). We established 32 primary cultures of colon cancer-associated fibroblasts derived from resected colon tumors from 26 patients as described previously [33]. The cells were cultured in DMEM supplemented with streptomycin (100 µg/ml), penicillin (100 U/ml) and 10% fetal bovine serum (FBS) at 37°C in a humidified environment of 90% air and 10% CO_2_. All the experiments were performed using cells cultured in medium supplemented with 10% FBS. All the tissue samples were obtained at the time of surgery at the Department of Surgery I, Kyushu University Hospital (Fukuoka, Japan). Experienced pathologists performed histologic examinations of all tissues adjacent to the specimens.

5-Fu and DOX were kindly provided by Wako Pure Chemical Industries (Osaka, Japan) and Nippon Roche K.K. (Kamakura, Japan), respectively.

### Flow cytometry

The tissue samples were washed extensively with phosphate-buffered saline (PBS), minced into approximately 1-mm^3^ cubes using scissors and dissociated into single cells by digestion with collagenase (Wako Pure Chemical Industries). The dissociated cells were passed through a filter. Primary cells derived from the resected tissues and dissociated DLD-1 cells were suspended in ice-cold 1% FBS in PBS at 1×10^6^ cells/100 µl. Each suspension was incubated with an antibody on ice for 40 min. The labeled cells were analyzed using an EPICS ALTRA flow cytometer (Beckman Coulter Inc., Fullerton, CA) equipped with two lasers that provided excitation wavelengths of 488 nm for the fluorescein isothiocyanate (FITC), phycoerythrin (PE) and propidium iodide (PI) signals and 635 nm for the allophycocyanin (APC) signals. The resulting fluorescence emissions were collected using band pass filter sets at 525±15 nm for FITC, 575±10 nm for PE, 610±12 nm for PI and 675±25 nm for APC. EXPO32 flow cytometry software (Beckman Coulter Inc.) was used to quantify the fluorescence signals and to set the logical electronic-gating parameters. The primary antibodies used for the FACS analyses are listed in Table S2.

### qRT-PCR

Total RNA was extracted from cultured and/or sorted cells using a High Pure RNA Isolation Kit (Roche Diagnostics, Mannheim, Germany) with DNase I (Roche Diagnostics) treatment, according to the manufacturer's instructions. We designed specific primers (Table S3) and performed BLAST searches to ensure the specificities of these primers. One-step qRT-PCR was performed using a QuantiTect SYBR Green Reverse Transcription-PCR Kit (Qiagen K.K., Tokyo, Japan) and a Chromo4 Real-Time PCR Detection System (Bio-Rad Laboratories, Hercules, CA), as described previously [34]. Each sample was run in triplicate and the expression of each gene was presented as the ratio between the expression of each target gene mRNA and that of *18S rRNA*.

### *CD10* siRNAs

Inhibition of *CD10* gene expression was achieved by RNA interference with siRNAs against *CD10* (siRNA-1: sense, 5′-GGUUGAAUUUCACAAAUGATT-3′, antisense, 5′-UCAUUUGUGAAAUUCAACCAG-3′; siRNA-2: sense, 5′-GUGUGGUGUGGAACCUAUATT-3′, antisense, 5′-UAUAGGUUCCACACCACACCT-3′; Qiagen, Poison Information Center Mainz, Germany) as described previously [35]. To verify the specificity of the knockdown effects, we used a control siRNA (Qiagen). Fibroblasts were transfected with 100 pmol of the appropriate siRNA using Nucleofector (Amaxa Biosystems, Köln, Germany) according to the manufacturer's instructions.

### *In vitro* invasion assay

The invasiveness of colon cancer cells was assessed based on the number of cells invading through Matrigel-coated transwell chambers (BD, Franklin Lakes, NJ) as described previously [36]. Briefly, transwell inserts with 8-µm pores were coated with Matrigel (20 µg/well; BD, Bedford, MA). Cells (5×10^4^ cells/ml) were seeded in the upper chambers. For cocultures, 5×10^4^ fibroblasts were seeded in the lower chambers. Thereafter, the cells were incubated for 48 h. Cells that invaded to the lower surface of the Matrigel-coated membrane were counted in five random fields at 200× magnification under a light microscope (ECLIPSE TE2000; Nikon, Tokyo, Japan). The results were expressed as the mean number of invading cells. Each experiment was carried out in triplicate wells, and independent experiments were repeated more than three times.

### Cell proliferation assay

Cell proliferation was evaluated by measuring the fluorescence intensity of PI, as described previously [36]. All experiments were performed in triplicate wells and repeated more than three times.

### *In vivo* experiments

Sorted DLD-1 or HCT116 colon cancer cells and cancer-associated fibroblasts were suspended in 100 µl of DMEM supplemented with 10% FBS, and subcutaneously transplanted into the limbs of 18 5-week-old female NOD/SCID mice (**NOD.CB17-Prkdc^scid^/shiJic**; Kyudo Co. Ltd., Tosu, Japan) [37]. Tumor appearance was evaluated using a caliper, and tumor volume was calculated using the formula π/6×(L×W×W), where L represents the largest tumor diameter and W represents the smallest tumor diameter. After different times of tumor growth, the animals were sacrificed.

### Microarray analysis

We performed microarray analyses using CD10^+^ and CD10^−^ primary colon fibroblasts. The qualities of the RNA samples were evaluated using a 2100 Bioanalyzer (Agilent Technologies, Waldbronn, Germany) as previously described [38]. We used a HumanWG-6 Expression BeadChip (Illumina, San Diego, CA) for these analyses. The data were analyzed using BeadStudio software version 3.2.3 (Illumina). All of the microarray data were deposited in CIBEX under the accession number CBX125 (http://cibex.nig.ac.jp/index.jsp).

### Statistical analyses

Statistical analyses were performed using JMP version 7.0.1 software (SAS Institute, Cary, NC). The CD133 and CD10 expressions were split into high- and low-level groups using recursive descent partition analysis, as described by Hoffmann et al. [39]. All data were expressed as the mean ± SD. The significance of differences between groups was estimated by Student's *t*-test and/or Spearman rank correlation analysis and repeated-measures ANOVA. Differences were considered significant at *P*<0.05.

## Supporting Information

Figure S1 Proliferation assay for unsorted DLD-1 (left panel) and HCT116 (right panel) parental cells and sorted CD133+ and CD133− colon cancer cells. Cells (2×104) were seeded and evaluated at 2 and 4 days after seeding by PI assays. Data represent means ± SD.
(0.42 MB TIF)

Figure S2 Colony formation assays for CD133+ and CD133− DLD-1 cells. Cells were propagated to allow colony formation for 14 days. Data represent means ± SD.
(0.20 MB TIF)

Figure S3 Resistance of CD133+ and CD133− DLD-1 cells to the anticancer agents 5-Fu (left panel) and DOX (right panel). Cells (3×104) were seeded and evaluated after 72 h of 5-Fu or DOX treatment. Data represent the fold changes in the number of viable cells (day 3/day 0).
(0.39 MB TIF)

Figure S4 Resistance of CD133+ and CD133− HCT116 cells to the anticancer agents 5-Fu (left panel) and DOX (right panel). Cells (3×104) were seeded and evaluated after 72 h of 5-Fu or DOX treatment. Data represent the fold changes in the number of viable cells (day 3/day 0).
(0.38 MB TIF)

Figure S5 Differential expression profiling of CD10+ and CD10− fibroblasts (f1). The validated data obtained by qRT-PCR are consistent with the microarray data. P<0.05 for all data.
(0.25 MB TIF)

Figure S6 Expression profiling of the CD10 cell population. Upper panel, CD10 expression in f2 fibroblasts transfected with control or CD10-targeting siRNAs. Lower panel, Effect of inhibition of CD10 on the expressions of CD10, MMP3, ALDH1A1, GPNMB and IGFBP2 in CD10+ fibroblasts. Black bars: control siRNA; gray bars: CD10 siRNA-1; white bars: CD10 siRNA-2. The expressions of CD10, MMP3, ALDH1A1, GPNMB and IGFBP2 was normalized by the expression of 18S rRNA. Data represent means ± SD.
(0.53 MB TIF)

Table S1 (0.04 MB DOC)

Table S2 (0.03 MB DOC)

Table S3 (0.03 MB DOC)
