# Supplementary figures and images for: Prospectively Isolated Cancer-Associated CD10+ Fibroblasts Have Stronger Interactions with CD133+ Colon Cancer Cells than with CD133− Cancer Cells
Source: PLoS One. 2010 Aug 12;5(8):e12121. doi: 10.1371/journal.pone.0012121 (PMC2920818; doi:10.1371/journal.pone.0012121)

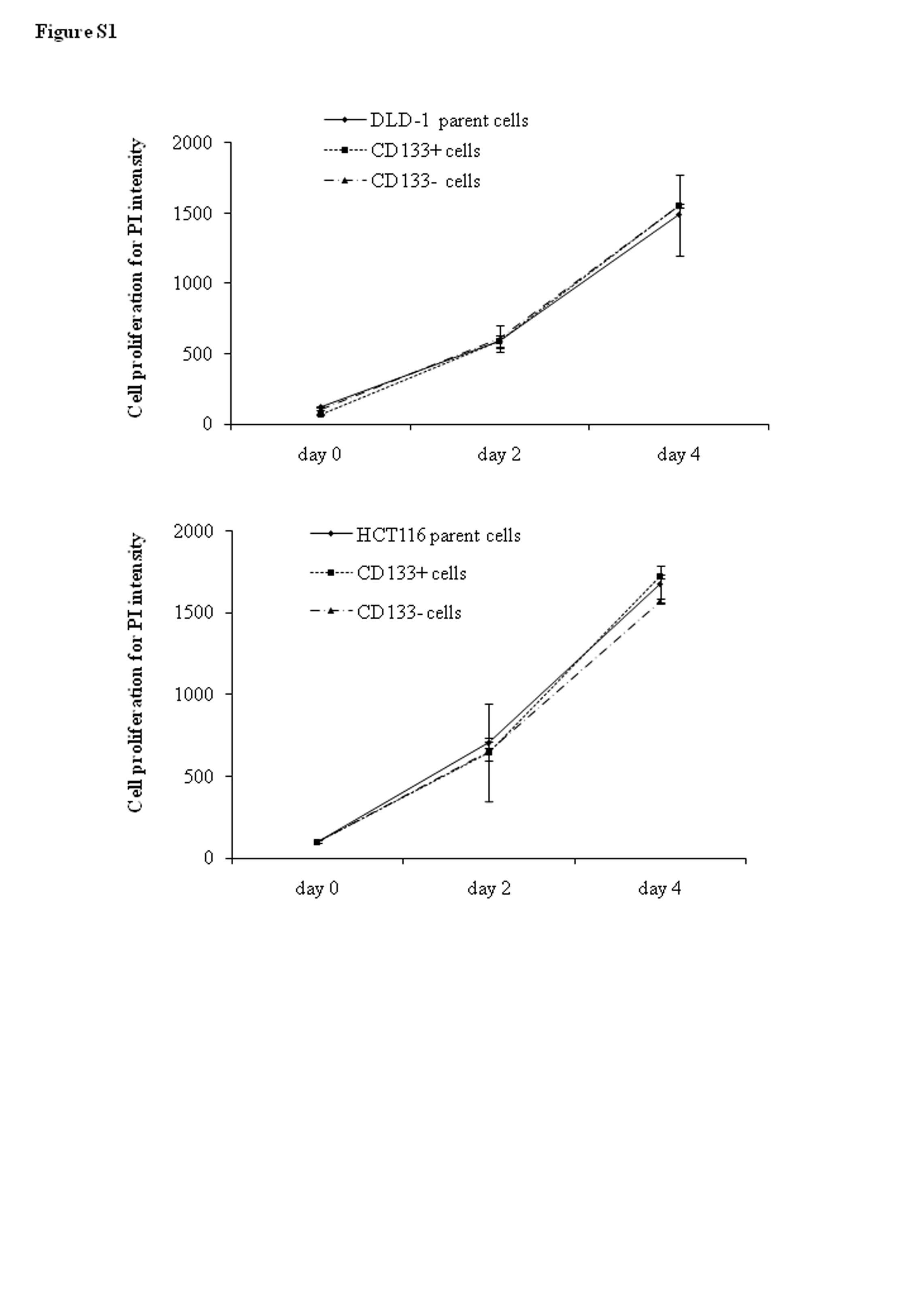

Supplement: Figure S1 — Proliferation assay for unsorted DLD-1 (left panel) and HCT116 (right panel) parental cells and sorted CD133+ and CD133− colon cancer cells. Cells (2×10^4^) were seeded and evaluated at 2 and 4 days after seeding by PI assays. Data represent means ± SD. (0.42 MB TIF) [file pone.0012121.s001.tif]

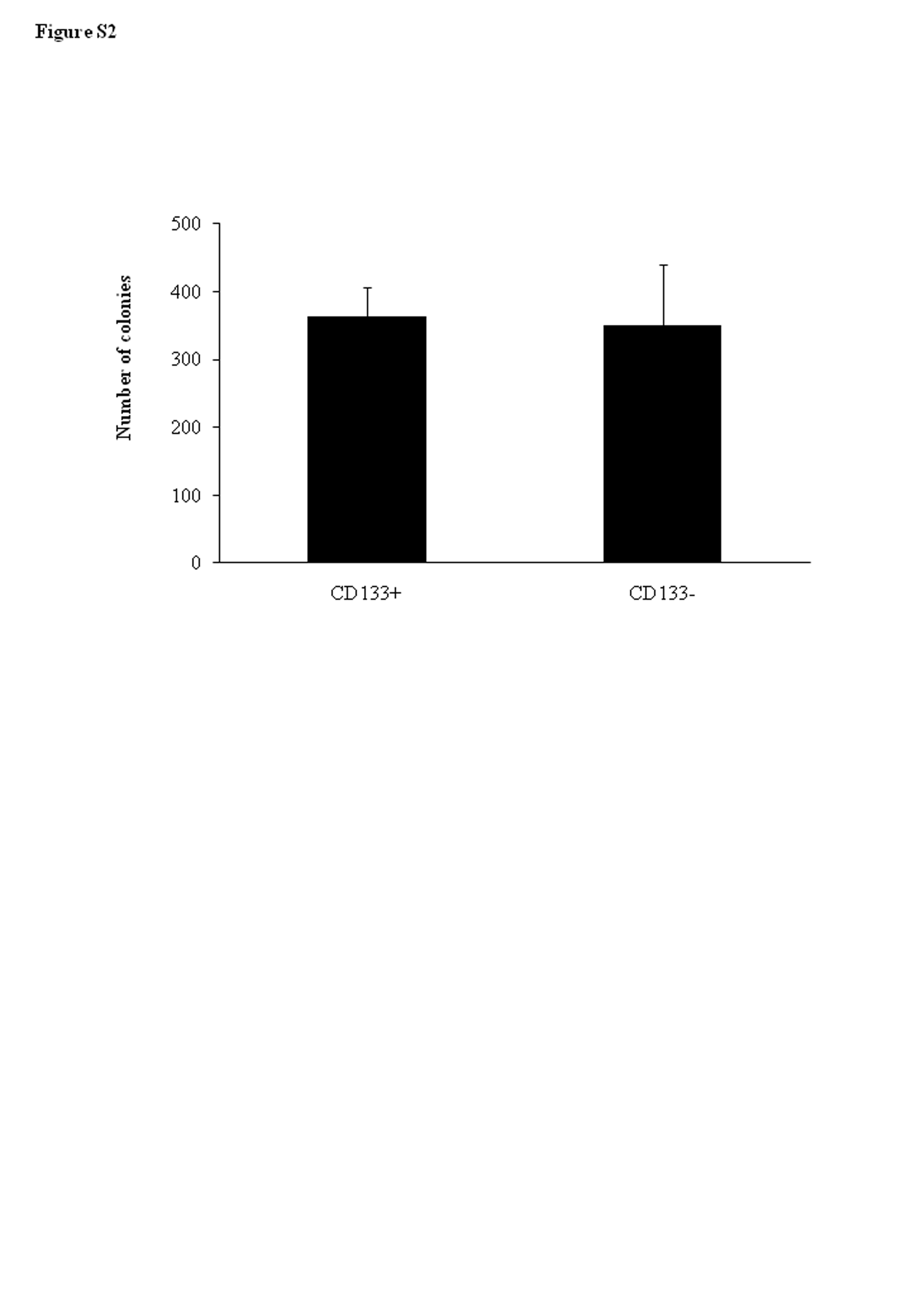

Supplement: Figure S2 — Colony formation assays for CD133+ and CD133− DLD-1 cells. Cells were propagated to allow colony formation for 14 days. Data represent means ± SD. (0.20 MB TIF) [file pone.0012121.s002.tif]

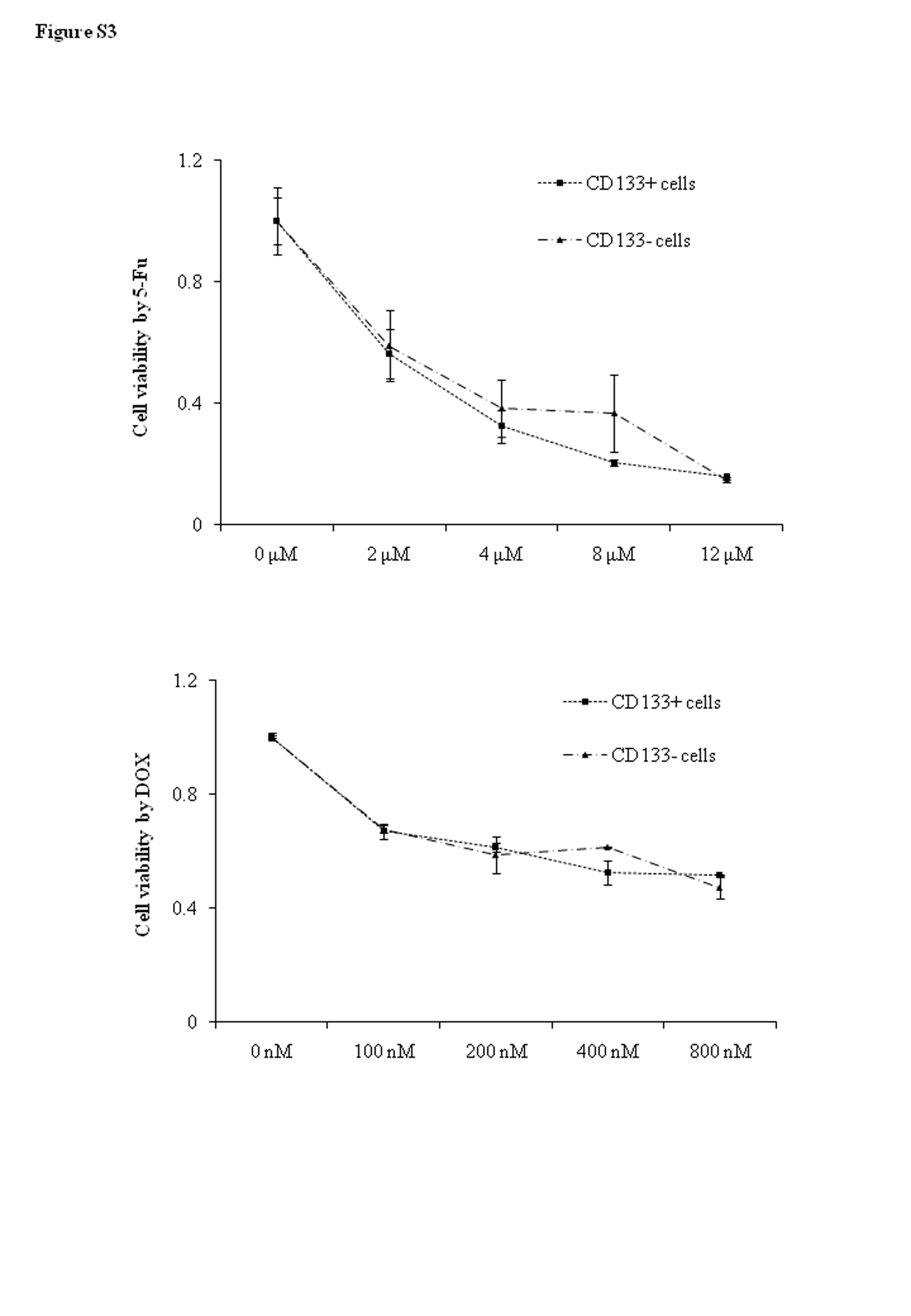

Supplement: Figure S3 — Resistance of CD133+ and CD133− DLD-1 cells to the anticancer agents 5-Fu (left panel) and DOX (right panel). Cells (3×10^4^) were seeded and evaluated after 72 h of 5-Fu or DOX treatment. Data represent the fold changes in the number of viable cells (day 3/day 0). (0.39 MB TIF) [file pone.0012121.s003.tif]

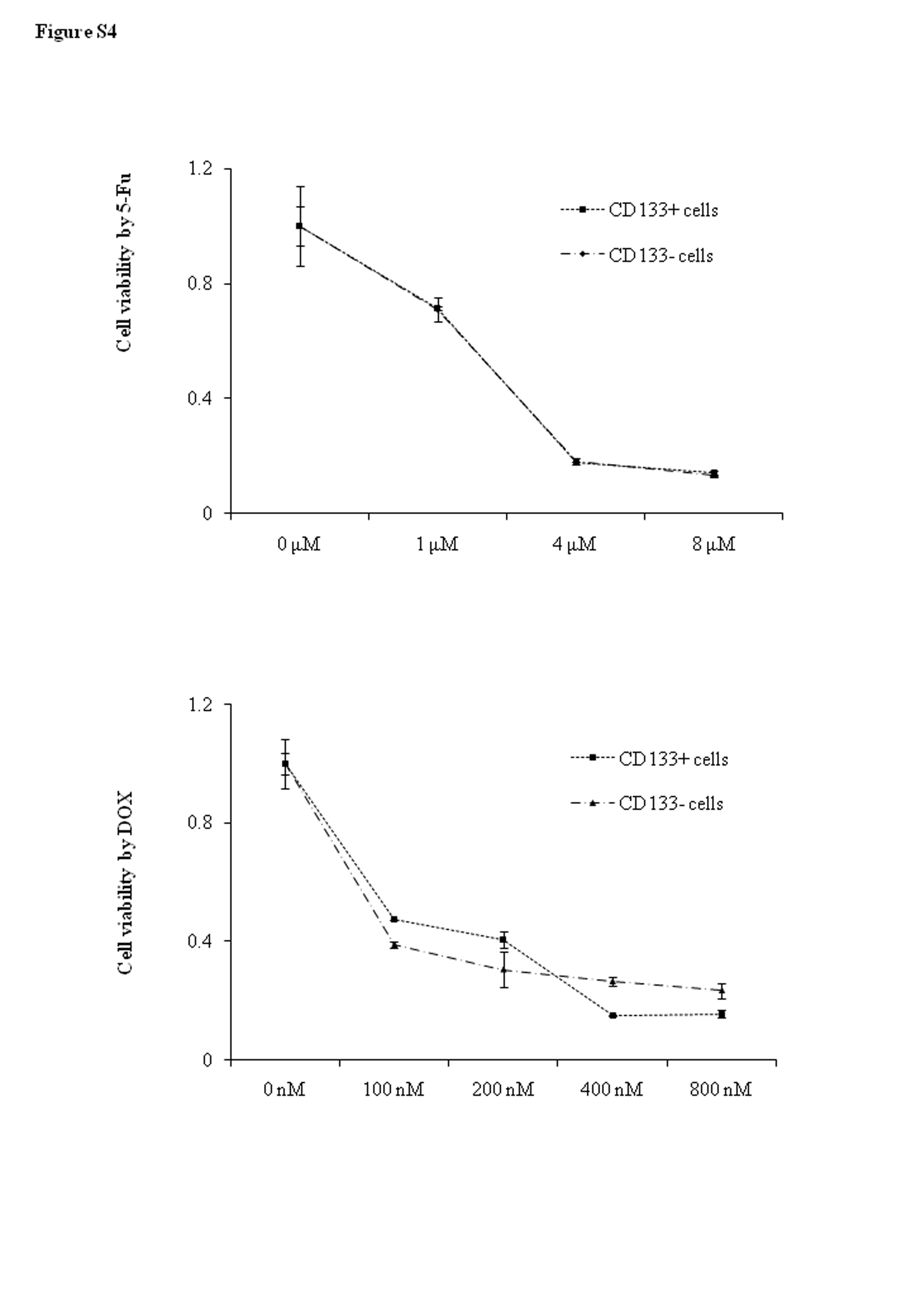

Supplement: Figure S4 — Resistance of CD133+ and CD133− HCT116 cells to the anticancer agents 5-Fu (left panel) and DOX (right panel). Cells (3×10^4^) were seeded and evaluated after 72 h of 5-Fu or DOX treatment. Data represent the fold changes in the number of viable cells (day 3/day 0). (0.38 MB TIF) [file pone.0012121.s004.tif]

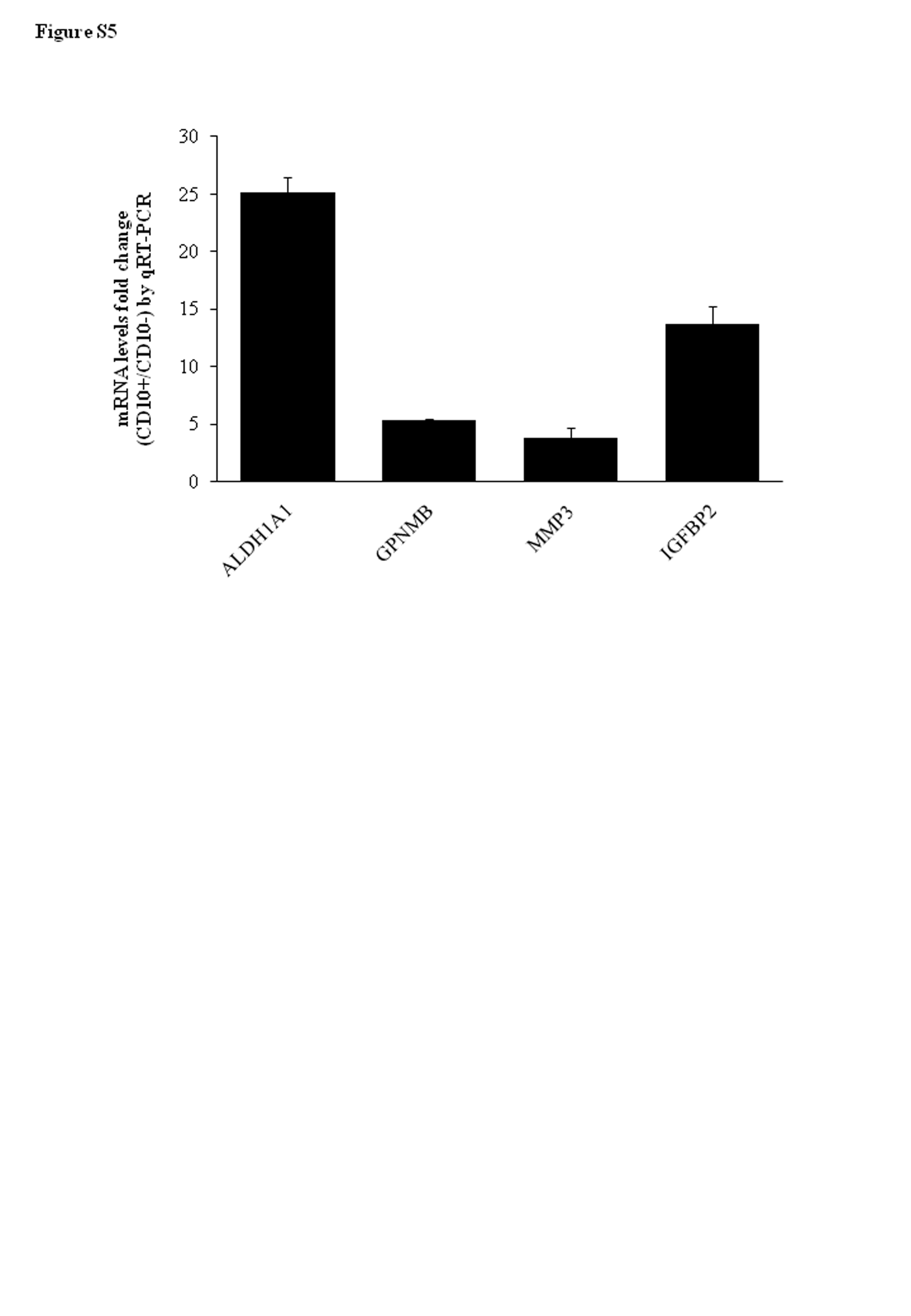

Supplement: Figure S5 — Differential expression profiling of CD10+ and CD10− fibroblasts (f1). The validated data obtained by qRT-PCR are consistent with the microarray data. P<0.05 for all data. (0.25 MB TIF) [file pone.0012121.s005.tif]

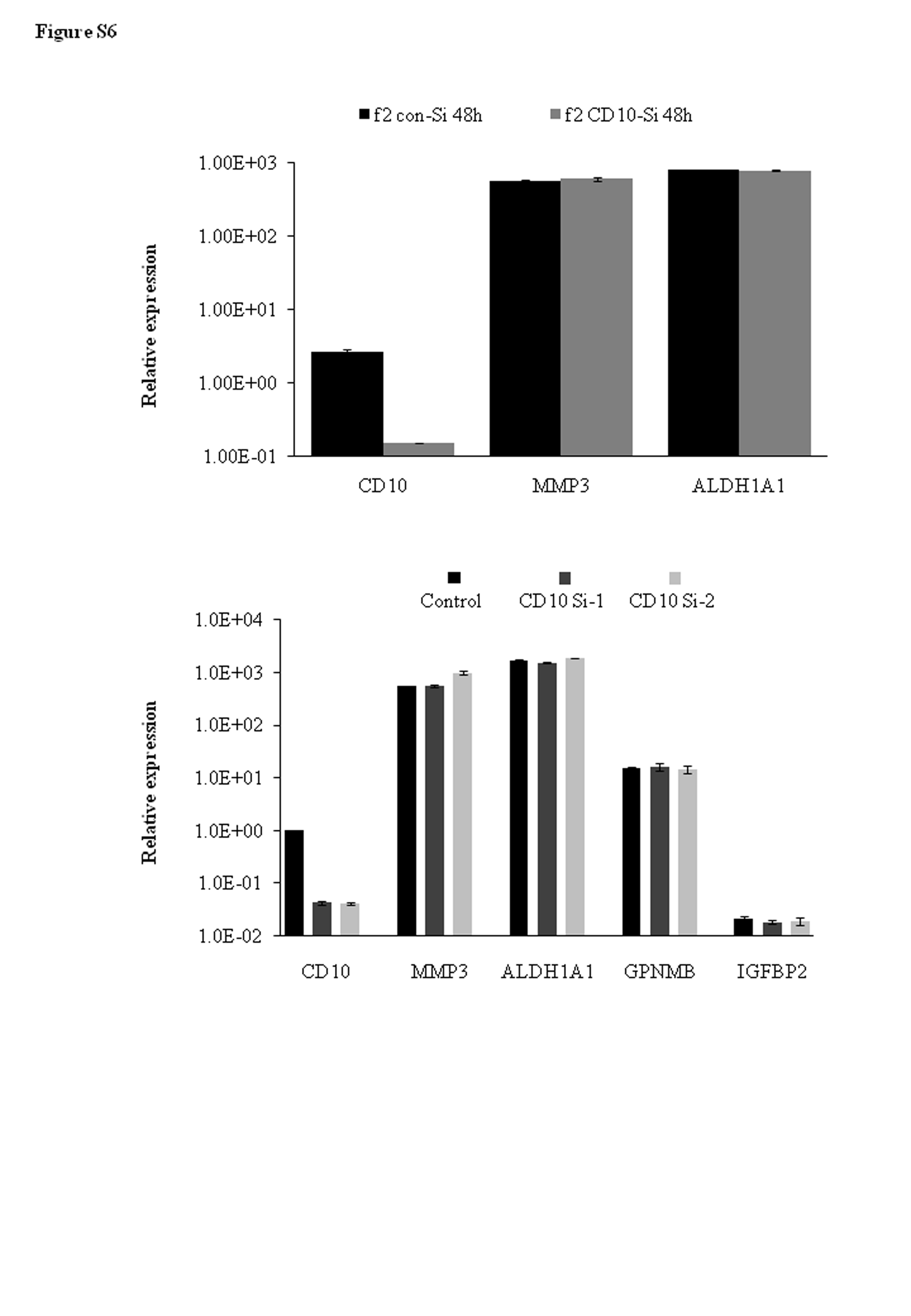

Supplement: Figure S6 — Expression profiling of the CD10 cell population. Upper panel, CD10 expression in f2 fibroblasts transfected with control or CD10-targeting siRNAs. Lower panel, Effect of inhibition of CD10 on the expressions of CD10, MMP3, ALDH1A1, GPNMB and IGFBP2 in CD10+ fibroblasts. Black bars: control siRNA; gray bars: CD10 siRNA-1; white bars: CD10 siRNA-2. The expressions of CD10, MMP3, ALDH1A1, GPNMB and IGFBP2 was normalized by the expression of 18S rRNA. Data represent means ± SD. (0.53 MB TIF) [file pone.0012121.s006.tif]
