# Supplementary material for: Prospectively Isolated Cancer-Associated CD10+ Fibroblasts Have Stronger Interactions with CD133+ Colon Cancer Cells than with CD133− Cancer Cells
Source: PLoS One. 2010 Aug 12;5(8):e12121. doi: 10.1371/journal.pone.0012121 (PMC2920818; doi:10.1371/journal.pone.0012121)
Supplement: Table S1 — (0.04 MB DOC) [file pone.0012121.s007.doc]

**Table S1**. Genes with fold change (CD10^+^/CD10^-^ >2)

| **Target ID** | **Accession** | **Definition** | **Fold change** |
| --- | --- | --- | --- |
| ALDH1A1 | NM_000689.3 | aldehyde dehydrogenase 1 family, member A1 | 4.95 |
| TUBB2B | NM_178012.3 | tubulin, beta 2B (TUBB2B) | 3.98 |
| CAMK2N1 | NM_018584.5 | calcium/calmodulin-dependent protein kinase II | 3.93 |
| GPNMB | NM_001005340.1 | glycoprotein (transmembrane) nmb (GPNMB), transcript variant 1 | 3.54 |
| LOC404266 | XR_001389.1 | hypothetical LOC404266 (LOC404266), misc RNA | 3.27 |
| MKX | NM_173576.1 | mohawk homeobox (MKX) | 3.25 |
| ACTC1 | NM_005159.4 | alpha, cardiac muscle 1 (ACTC1) | 3.17 |
| MMP3 | NM_002422.3 | matrix metallopeptidase 3 (stromelysin 1, progelatinase) (MMP3) | 3.11 |
| A2M | NM_000014.4 | alpha-2-macroglobulin (A2M) | 2.87 |
| IGFBP2 | NM_000597.2 | insulin-like growth factor binding protein 2, 36kDa (IGFBP2) | 2.76 |
| LBH | NM_030915.1 | limb bud and heart development homolog (mouse) (LBH) | 2.52 |
| STMN2 | NM_007029.2 | stathmin-like 2 (STMN2) | 2.37 |
| SPINK6 | NM_205841.2 | serine peptidase inhibitor, Kazal type 6 (SPINK6) | 2.29 |
| ELN | NM_001081754.1 | elastin (supravalvular aortic stenosis, Williams-Beuren syndrome) (ELN), transcript variant 4 | 2.22 |
| DMN | NM_015286.5 | desmuslin (DMN), transcript variant B | 2.21 |
| SERPINB7 | NM_003784.2 | serpin peptidase inhibitor, clade B (ovalbumin), member 7 (SERPINB7), transcript variant 1 | 2.2 |
| TNS3 | NM_022748.10 | tensin 3 (TNS3) | 2.2 |
| GPR124 | NM_032777.6 | G protein-coupled receptor 124 (GPR124) | 2.13 |
| HSPB7 | NM_014424.3 | heat shock 27kDa protein family, member 7 (cardiovascular) | 2.11 |
| HSPA2 | NM_021979.2 | heat shock 70kDa protein 2 (HSPA2) | 2.08 |

*CD10^+^ and CD10^-^ *P* < 0.05
