# Supplementary material for: Prospectively Isolated Cancer-Associated CD10+ Fibroblasts Have Stronger Interactions with CD133+ Colon Cancer Cells than with CD133− Cancer Cells
Source: PLoS One. 2010 Aug 12;5(8):e12121. doi: 10.1371/journal.pone.0012121 (PMC2920818; doi:10.1371/journal.pone.0012121)
Supplement: Table S2 — (0.03 MB DOC) [file pone.0012121.s008.doc]

**Table S2**. Antibodies used for flow cytometry

| **Antibodies** | **Labeled** | **Maker** |
| --- | --- | --- |
| CD133/2 (293C3) | PE | MACS |
| CD24 | PE | eBioscience |
| CXCR4 | PE | R&D |
| CD326 (EpCAM) | APC | MACS |
| C-Kit | PE | eBioscience |
| CD44 | FITC | MBL |
| CD10 | PE | Bioscence |
| CD105 | FITC | R&D |
| CD54 | PE | Beckman Coulter |
| CD31 | FITC | MACS |
| CD45 | APC | MACS |
