# Supplementary material for: Prospectively Isolated Cancer-Associated CD10+ Fibroblasts Have Stronger Interactions with CD133+ Colon Cancer Cells than with CD133− Cancer Cells
Source: PLoS One. 2010 Aug 12;5(8):e12121. doi: 10.1371/journal.pone.0012121 (PMC2920818; doi:10.1371/journal.pone.0012121)
Supplement: Table S3 — (0.03 MB DOC) [file pone.0012121.s009.doc]

**Table S3**. Primers used for real time RT-PCR

| **Primer** | **Forward Sequence 5’-3’** | **Reverse Sequence 5’-3’** |
| --- | --- | --- |
| *CD133* | GCCACCGCTCTAGATACTGC | GCTTTTCCTATGCCAAACCA |
| *CD10* | CCTTCTTTAGTGCCCAGCAG | TGAGTCCACCAGTCAACGAG |
| *CD105* | GTGACGGTGAAGGTGGAACT | GATCTGCATGTTGTGGTTGG |
| *CD44* | AGAAGGTGTGGGCAGAAGAA | AAATGCACCATTTCCTGAGA |
| *ALDH1A1* | TTGGAATTTCCCGTTGGTTA | TGCTCTGCTGGTTTGACAAC |
| *GPNMB* | CATCATTTCTGACCCCACCT | GGTTCGTCTCACAGTCAGCA |
| *MMP3* | GAAGCTGGACTCCGACACTC | GATGCCAGGAAAGGTTCTGA |
| *IGFBP2* | AGCATGGCCTGTACAACCTC | GGTGTTGGGGTTCACACAC |
| *18S r*RNA | GTAACCCGTTGAACCCCATT | CCATCCAATCGGTAGTAGCCG |
